# Carbapenemase genes and mortality in patients with carbapenem-resistant Enterobacterales, Atlanta, Georgia, 2011–2020

**DOI:** 10.1017/ash.2023.226

**Published:** 2023-09-29

**Authors:** Lucy Witt, Ahmed Babike, Gillian Smith, Sarah Satola, Mary Elizabeth Sexton, Jesse Jacob

## Abstract

**Background:** Carbapenemase genes in carbapenem-resistant Enterobacterales (CP-CRE) may be transmitted between patients and bacteria. Reported rates of carbapenemase genes vary widely, and it is unclear whether having a carbapenemase gene portends worse outcomes given that all patients with CRE infections have limited treatment options. **Methods:** Using active population- and laboratory-based active surveillance data collected by the US CDC-funded Georgia Emerging Infections Program from 2011 to 2020, we assessed the frequency of carbapenemase genes in a convenience sample of CRE isolates using whole-genome sequencing (WGS), and we investigated risk factors for carbapenemase positivity. Only the first isolate per patient in a 30-day period was included. We compared characteristics of patients with CP-CRE and non–CP-CRE. Using multivariable log binomial regression, we assessed the association of carbapenemase gene positivity and 90-day mortality. **Results:** Of 284 CRE isolates, 171 isolates (60.2%) possessed a carbapenemase gene (Table 1), and KPC-3 was the most common carbapenemase gene (80.7%), with only 7 isolates possessing NDM (Table 2). No isolates possessed >1 carbapenemase gene, and most isolates were from urine (82.4%) (Table 1). Carbapenemase gene positivity was associated with lower age, male sex, black race, infection with *Klebsiella pneumoniae*, polymicrobial infection, having an indwelling medical device, receiving chronic dialysis, and prior stay in a long-term acute-care hospital, long-term care facility, and/or prior hospitalization in the last year. The 90-day mortality rates were similar in patients with non–CP-CRE and CP-CRE: 24.8% versus 25.7% (*P* = .86). In multivariable analysis, carbapenemase gene presence was not associated with 90-day mortality (adjusted risk ratio, 0.82; 95% CI, 0.50–1.35) when adjusting for CCI, infection with *Klebsiella pneumoniae*, and chronic dialysis use. **Conclusions:** The frequency of CP-CRE among CRE was high in this study, but unlike prior studies, the 90-day mortality rates wer similar in patients with CP-CRE compared to non–CP-CRE. Our results provide novel associations (eg, lower age, male sex, infection with *Klebsiella pneumoniae*, and indwelling medical devices) that infection preventionists could use to target high-risk patients for screening or isolation prior to CP-CRE detection.

**Figure:**
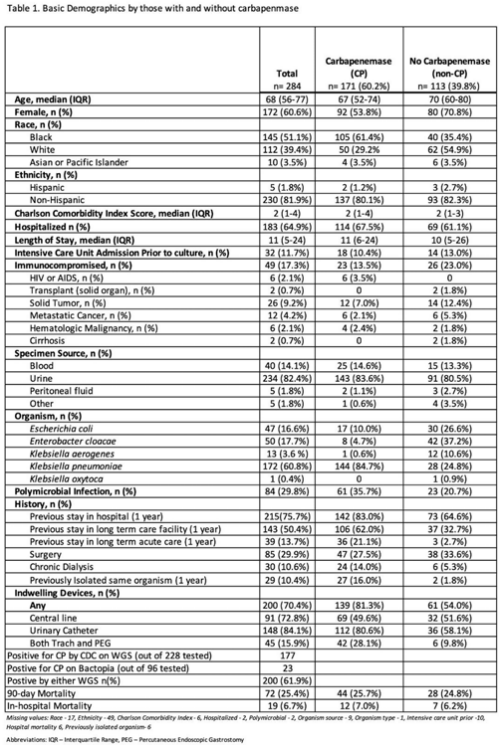

**Figure:**
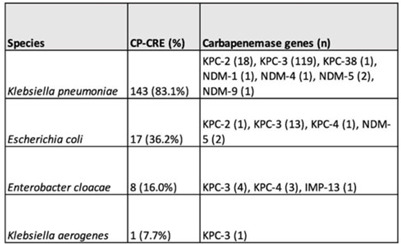

**Disclosure:** None

